# *Pseudomonas aeruginosa* Keratitis in Mice: Effects of Topical Bacteriophage KPP12 Administration

**DOI:** 10.1371/journal.pone.0047742

**Published:** 2012-10-17

**Authors:** Ken Fukuda, Waka Ishida, Jumpei Uchiyama, Mohammad Rashel, Shin-ichiro Kato, Tamae Morita, Asako Muraoka, Tamaki Sumi, Shigenobu Matsuzaki, Masanori Daibata, Atsuki Fukushima

**Affiliations:** 1 Department of Ophthalmology and Visual Science, Kochi Medical School, Kochi, Japan; 2 Department of Microbiology and Infection, Kochi Medical School, Kochi, Japan; 3 Research Institute of Molecular Genetics, Kochi University, Kochi, Japan; 4 Kochi Medical School Hospital, Kochi, Japan; 5 Kochi Gakuen Junior College, Kochi, Japan; Charité, Campus Benjamin Franklin, Germany

## Abstract

The therapeutic effects of bacteriophage (phage) KPP12 in *Pseudomonas aeruginosa* keratitis were investigated in mice. Morphological analysis showed that phage KPP12 is a member of the family *Myoviridae*, morphotype A1, and DNA sequence analysis revealed that phage KPP12 is similar to PB1-like viruses. Analysis of the phage KPP12 genome did not identify any genes related to drug resistance, pathogenicity or lysogenicity, and so phage KPP12 may be a good candidate for therapeutic. KPP12 showed a broad host range for *P. aeruginosa* strains isolated from clinical ophthalmic infections. Inoculation of the scarified cornea with *P. aeruginosa* caused severe keratitis and eventual corneal perforation. Subsequent single-dose administration of KPP12 eye-drops significantly improved disease outcome, and preserved the structural integrity and transparency of the infected cornea. KPP12 treatment resulted in the suppression of neutrophil infiltration and greatly enhanced bacterial clearance in the infected cornea. These results indicate that bacteriophage eye-drops may be a novel adjunctive or alternative therapeutic agent for the treatment of infectious keratitis secondary to antibiotic-resistant bacteria.

## Introduction

Bacteriophages (phages) are viruses that are omnipresent in the environment (including water, soil, food, and the gastrointestinal tract) and which were discovered more than 80 years ago [1], [2]. Phages infect, lyse, and kill bacteria without damage to mammalian cells. Thus, phage therapy was immediately considered a potential treatment for bacterial infectious diseases. However, the introduction of antibiotic therapy meant that phage therapy was restricted to countries in Eastern Europe and the former Soviet Union. Since the 1980s, phage therapy has been re-evaluated in Western countries due to the emergence of antibiotic resistance [3]. The therapeutic efficacy of phage therapy *in vivo* has been demonstrated in several murine models of antibiotic-resistant bacterial infection, including methicillin-resistant *Staphylococcus aureus* (MRSA), vancomycin-resistant *Enterococcus* (VRE), and multidrug-resistant *Pseudomonas aeruginosa* (*P. aeruginosa*) and *Escherichia coli* [4], [5], [6], [7]. In addition, results from clinical studies into bacteriophage treatment in humans, which have been performed mainly in Eastern Europe, have been reported [8], [9], [10]. Few studies have investigated phage therapy for ocular diseases, and these have mainly focused on conjunctivitis [8], [11].

The cornea is an avascular and transparent tissue that acts as a lens. Its transparency is primarily attributable to the architecture of corneal stromal collagen. Infectious keratitis is a sight-threatening disease that requires prompt diagnosis and appropriate treatment if tissue damage and scarring are to be prevented. *P. aeruginosa* is an important cause of destructive ocular infection, especially in contact lens wearers [12]. *P. aeruginosa* keratitis progresses rapidly and is characterized by the infiltration of inflammatory cells and tissue destruction, which can result in corneal perforation. Standard treatment is the administration of antibiotics to eliminate the infectious organisms. In recent years, however, antibiotic-resistant bacterial infections such as MRSA, multidrug-resistant *P. aeruginosa,* and VRE have emerged as a major clinical problem. The ocular surface is a common site of infection for multidrug-resistant bacteria such as MRSA [13], [14]. The development of new adjunctive or alternative therapies for the treatment of bacterial keratitis is therefore warranted.

The aim of the present study was to characterize *P. aeruginosa*-specific bacteriophage KPP12 and investigate the effects of single-dose administration of KPP12 eye-drops on *P. aeruginosa* keratitis in mice.

## Materials and Methods

### Ethical Treatment of Animals

This study was approved by the Committee for Care and Use of Laboratory Animals at Kochi University (Permit Number: H23-070/E-00058) and was carried out in strict accordance with the Association for Research in Vision and Ophthalmology Statement on the Use of Animals in Ophthalmic and Vision Research. The animals were treated humanely and all efforts were made to minimize suffering.

### Bacterial Strains, Reagents and Culture Media

All bacterial strains used in the present study are listed in Table 1. The *P. aeruginosa* strains PA21 and PA33 were used for phage preparation and the infection experiments, respectively. All chemicals and reagents were purchased from Nacalai (Kyoto, Japan) and Sigma-Aldrich (St. Louis, MO), unless otherwise stated. Luria-Bertani (LB) medium was used for bacteria or phage culture. M9 medium was used for phage dilution. Difco *Pseudomonas* isolation agar (Becton, Dickinson and Co., Sparks, MD) was used for counting colony forming units (CFU) of *P. aeruginosa* harvested from eye tissue. Culture media for phage preparation were purchased from Becton, Dickinson and Co. During phage-plaque formation, a LB medium containing 1.5% or 0.5% agar was used for the lower and the upper layer, respectively.

**Table 1:** *P. aeruginosa* strains used in the present study and their sensitivity to phage KPP12.

Strain*	Sensitivity to KPP12**	Department of Origin	Material
PA1	+	Neurosurgery	Sputum
PA2	+	Surgery	Pus
PA3	−	Internal Medicine	Sputum
PA4	−	Internal Medicine	Sputum
PA5	L	Surgery	Pus
PA6	+	Internal Medicine	Sputum
PA7	+	Otolaryngology	Pus
PA8	+	Internal Medicine	Sputum
PA9	L	Otolaryngology	Pus
PA10	+	Internal Medicine	Other
PA11	−	Pediatrics	Other
PA12	L	Pediatrics	Pharynx
PA13	+	Otolaryngology	Pus
PA14	−	Urology	Urine
PA15	−	Urology	Catheter
PA16	−	Surgery	Intestine
PA17	+	Surgery	Pus
PA18	+	Surgery	Pus
PA19	+	Urology	Sputum
PA20	−	Internal Medicine	Urine
PA21	+	Dermatology	Pus
PA22	L	Pediatrics	Sputum
PA23	−	Pediatrics	Pharynx
PA24	+	Otolaryngology	Sputum
PA25	−	Surgery	Sputum
PA26	+	Internal Medicine	Sputum
PA27	−	Otolaryngology	Pus
PA28	−	Dermatology	Pus
PA29	−	Internal Medicine	Blood
PA30	+	Internal Medicine	Sputum
PA31	+	Ophthalmology	Pus
PA32	+	Ophthalmology	Pus
PA33	+	Ophthalmology	Pus
PA34	+	Ophthalmology	Pus
PA35	L	Ophthalmology	Pus
PAO1	+		
D4	+		
S10	L		

* PA1 to PA35, Kochi Medical School Hospital; PAO1, D4, and S10, Dr. Matsumoto, Tokyo Medical University.

** +, plaque formation; −, no plaque formation; L, lysis from without.

### Isolation and Host Specificity of Phage KPP12

Phage KPP12 was isolated from a water sample using a *P. aeruginosa* strain (strain PA21) as the host. The water sample had been collected from a river in Kochi prefecture, Japan. An inoculation loop was used to streak a drop of phage (ca. 10 µl) onto double-layer agar containing a *P. aeruginosa* strain. This was then incubated at 37°C overnight. After incubation, the presence of a plaque or broad diffusible translucent areas indicated sensitivity to phage KPP12 (lysis from within). No plaques/traces or a semi-translucent trace that disappeared gradually indicated no sensitivity to phage KPP12 or lysis from without, respectively.

### Large-scale Culture and Purification of *P. aeruginosa* Phages

Phage was cultured with *P. aeruginosa* strain PA21 in 300 ml of LB. After complete bacterial lysis, the phage lysate was collected by centrifugation, and polyethylene glycol 6000 and NaCl were added to final concentrations of 10% and 0.5 M, respectively. The mixture was stored at 4°C overnight, and the phage pellet was obtained by centrifugation (10,000×*g*, 20 min, 4°C). The phage pellet was dissolved in TM buffer (10 mM Tris-HCl [pH 7.2], 5 mM MgCl_2_) containing 50 µg/ml of DNase I and RNase A and then incubated at 37°C for 1 h. The phage was then purified by density gradient ultracentrifugation using either CsCl or iodixanol as the centrifugal medium.

For the non-animal experiments, the phage suspension was placed on top of a discontinuous CsCl gradient (ρ = 1.3, 1.5, and 1.7) and centrifuged (100,000×*g*, 1 h, 4°C). After phage band collection, the phage band was again purified by CsCl density gradient ultracentrifugation. The phage band was collected and dialyzed against 0.1 M ammonium acetate, 10 mM NaCl, 1 mM CaCl_2_, and 1 mM MgCl_2_ (pH 7.2; AAS) for 1 h at 4°C. The phage concentration was measured by plaque assay.

For the animal experiments, phages were purified by iodixanol density gradient ultracentrifugation. Iodixanol ultracentrifugation medium was purchased from AXIS-SHIELD PoC AS (OptiPrep™, Oslo, Norway). Iodixanol was diluted to the appropriate concentration using saline. The phage suspension was then placed on top of a discontinuous iodixanol gradient (30%, 35%, and 40%) and centrifuged (200,000×*g*, 2 h, 4°C). After collection of the phage band, iodixanol density gradient ultracentrifugation was repeated. The phage band was collected and stored at 4°C until use. The concentration of the purified phage was measured by plaque assay.

### Genome Sequence Analysis

The phage in AAS was pelleted by ultracentrifugation (100,000×*g*, 1 h, 4°C). The phage pellet was then treated with 1% SDS for 10 min at 37°C, and phage DNA was extracted with water-saturated phenol. The DNA was washed twice in 70% ethanol prior to preparation. Finally, the phage DNA was dissolved in sterile distilled water. The genome was digested by EcoRI (Takara Bio, Shiga, Japan), and shotgun clones were prepared using plasmid pUC18. Primers were designed on the basis of the clone sequences, and the genome sequence was determined by genome direct sequencing using the primer walking method. Both strands were sequenced (at least 2-fold redundancy). Assembly of the determined sequences was performed manually by comparing overlapping regions. All sequencing was performed using a BigDye Terminator v1.1 Cycle Sequencing Kit (Applied Biosystems, Foster City, CA, USA) according to the manufacturer’s instructions, and an ABI PRISM 3100-Avant Genetic Analyzer (Applied Biosystems). Putative open reading frames (ORFs) were determined manually, using the automatic prediction provided by the microbial gene-finding program Prodigal as a reference (http://prodigal.ornl.gov/) [15]. Putative tRNA genes were screened using tRNAscan-SE 1.21 (http://selab.janelia.org/tRNAscan-SE/) [16]. The genome sequence of phage KPP12 was deposited into Genbank (accession: AB560486). Amino acid sequences were compared using the protein Basic Local Alignment Search Tool (BLASTp) of the National Center for Biotechnology Information (NCBI). The protein domain was predicted from a Conserved Domain Database (CDD) search using the NCBI website (http://www.ncbi.nlm.nih.gov/Structure/cdd/wrpsb.cgi). The results of the CDD search and BLASTp were used to predict the function of the ORF.

### Structural Protein Analysis of Phage KPP12

After dissolving the purified phage pellet into AAS and adding an equal volume of 2× Laemmli sample buffer, the sample was boiled for 5 min. The phage proteins were separated using a 12.5% SDS-polyacrylamide gel electrophoresis (PAGE) gel. After electrophoresis, the gel was stained with Coomassie brilliant blue R-250.

After SDS-PAGE, the phage proteins were blotted onto a polyvinylidene difluoride (PVDF) membrane (Sequi-Blot PVDF Membrane, Bio-Rad Laboratories) using a blotting solution (10 mM CHAPS [pH 11], 10% methanol), and a Hoefer TE70 semi-dry transfer unit (Hoefer, San Francisco, CA) at 1.2 cm^2^/mA for 90 min at 4°C. The blotted membrane was stained with Coomassie brilliant blue R-250. The target protein band was excised from the membrane and analyzed using a protein sequencer PPSQ-31A/33A (Shimadzu Biotech, Kyoto, Japan).

### Electron Microscopy

The purified phage sample in AAS was loaded onto a formvar-carbon coated copper membrane and negatively stained with 2% uranyl acetate (pH 4.0). Electron micrograph images were obtained with a Hitachi H-7100 transmission electron microscope (Hitachi) at 100 kV.

### Mouse Model of *P. aeruginosa* Keratitis

Specific-pathogen-free female C57BL/6 mice were purchased from Japan SLC Inc. (Shizuoka, Japan) and housed under pathogen-free conditions at the animal facility of Kochi Medical School. *P. aeruginosa* PA33 cells were grown in 30 ml LB medium at 37°C, and then centrifuged at 7,000 *g* for 5 min at the logarithmic growth phase (ca.100 Klett units). The cell pellet was washed with 30 ml saline, re-centrifuged under the same conditions, and then resuspended in ca. 3 ml saline. After appropriate dilution, turbidity (in Klett units) was measured to determine bacterial cell numbers. One Klett unit was assumed to be equivalent to 6.2×10^6^ *P. aeruginosa* cells/ml. This assumption was based on a previously standardized correlation between turbidity and bacterial cell numbers counted directly with a Petroff-Hausser counting chamber (Hausser Scientific, Suite C Horsham, PA).

The present mouse model of *P. aeruginosa* keratitis is described elsewhere 17]. Briefly, the cornea of the left eye in eight-week-old female C57BL/6 mice was then visualized under a stereoscopic microscope, and three 1 mm scratches were made using a sterile 25 gauge needle. A 5 μl aliquot containing 5×10^6^ cells of *P. aeruginosa* (PA33) was applied to the corneal surface. Thirty minutes after infection, 5×10^8^ plaque forming units (PFU) of phage in 5 μl or vehicle was applied to the corneal surface. The eyes were examined at 1, 3, and 5 days post-infection (p.i.) to grade disease severity according to an established scale, as described previously 18]. Strain PA33 was derived from a specimen obtained from a patient with *Pseudomonas* keratitis at the Kochi Medical School Hospital, Kochi, Japan.

### Determination of the Number of Viable Bacteria in the Infected Cornea

Mice were euthanized by inhalation of ether on day 5 p.i. and the corneas were harvested. Individual corneas were homogenized with a tissue homogenizer in sterile saline. Portions of the homogenized tissue samples were plated onto Difco *Pseudomonas* isolation agar after dilution in saline (either 1∶1, 1∶100, or 1∶10,000) and cultured at 37°C for 48 h to detect *P. aeruginosa* challenge. Results are reported as CFU per ml.

### Myeloperoxidase (MPO) Assay

A MPO assay was used to quantitate PMN number in the cornea, as described previously with slight modification [19]. Infected corneas were excised on day 5 p.i. and homogenized in 1.0 ml of 50 mM potassium phosphate (pH 6.0). The samples were then centrifuged. Pellets were resuspended in 0.03 ml of 50 mM phosphate buffer and 50 mM hexadecyltrimethylammonium bromide (Sigma-Aldrich), and 0.07 ml of 50 mM potassium phosphate was added. Then samples were freeze-thawed three times and centrifuged at 10,000 rpm for 10 min, and 0.02 ml of the supernatant was added to 0.05 ml substrate reagent (R&D systems, Minneapolis, MN) for 20 min at room temperature. Then 2N H_2_SO_4_ was added to the samples. Absorption was assessed by measuring the optical density at 450 nm.

### Histology and Immunostaining of PMN, *P. aeruuginosa*, and Collagen Fibrils

Eyes were harvested on day 5 p.i. and fixed in 4% formalin and embedded in paraffin. Sections were stained with hematoxylin/eosin, picrosirius red, and with the antibodies for granulocytes and *P. aeruginosa*. The antigen unmasking procedures involved heating with 10 mM citrate buffer, pH 6.0, for 15 min. Endogenous peroxidase activity was inhibited by adding 0.1% H_2_O_2_ in methanol for 15 min at room temperature. Sections were incubated with a rat anti-mouse Ly-6G and Ly6C antibody (1∶200; Becton, Dickinson and Co.) overnight at 4 °C. This was followed by incubation with a biotinylated rabbit anti-rat IgG antibody (1∶200; Dako Cytomation, Glostrup, Denmark) for 1 h. Sections were icubated using an Avidin-Biotin-Complex kit (Vector Laboratories, Burlingame, CA). *P. aeruginosa* was stained using mouse anti-*Pseudomonas aeruginosa* antibody (1∶50; Abcam, Cambridge, United Kingdom) and the Histofine mouse stain kit (Nichirei Biosciences Inc., Tokyo, Japan). Counterstaining was performed using Mayer’s Hematoxylin (Wako Pure Chemical Industries, Osaka, Japan). Control sections were processed in a similar manner but with the omission of the primary antibody. Staining of collagen type I and type III was performed using picrosirius red stain kit (Polysciences, Inc., Warrington, PA). Sections were photographed under a microscope (Biorevo; Keyence Corporation, Osaka, Japan).

### Statistical Analysis

The difference in the clinical scores between the two groups at each time point and the results of the MPO assays were tested using an unpaired, two-tailed Student’s *t*-test. The Mann-Whitney *U* test was used to determine the significance of viable bacterial counts. Data were considered significant at p<0.05.

## Results

### Characterization of Phage KPP12

**Figure 1:**
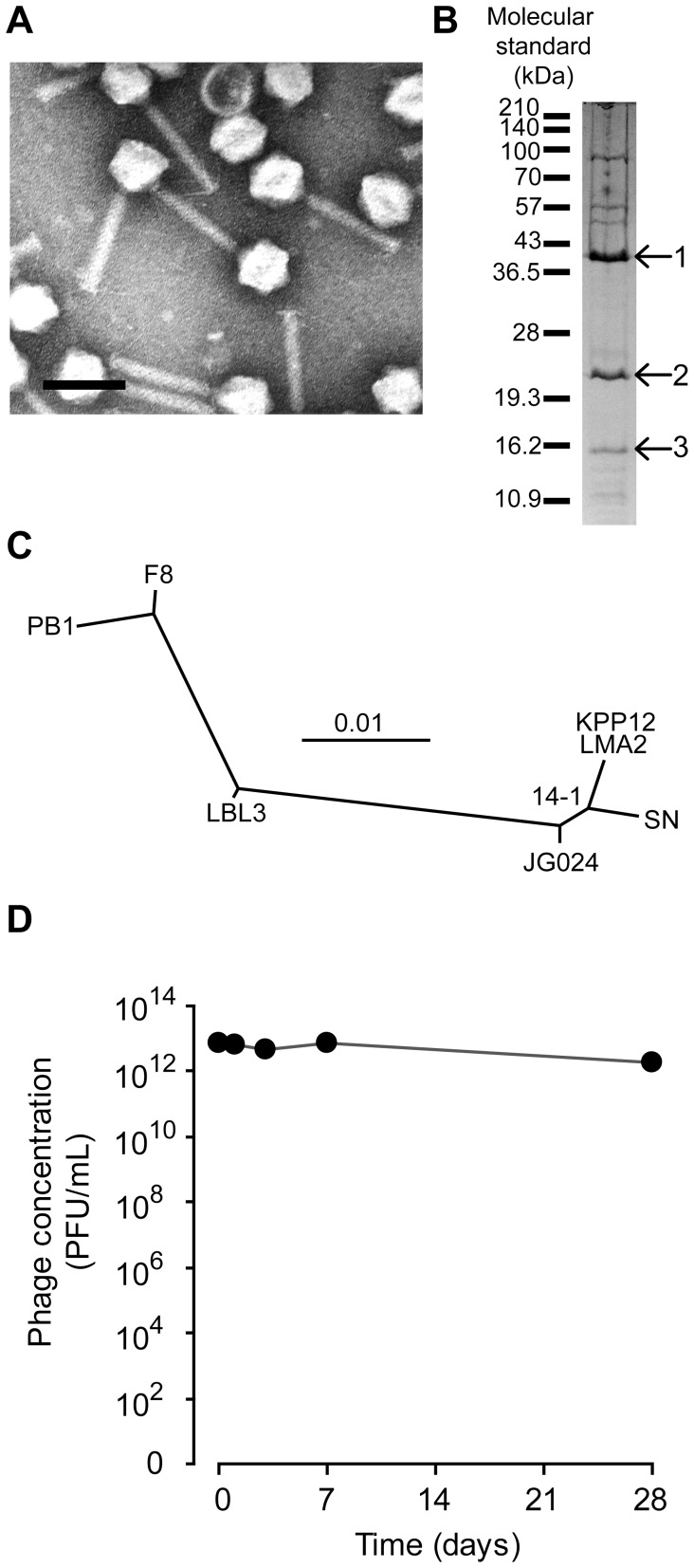
Characteristics of *P. aeruginosa* phage KPP12. (A) Morphology. The bar indicates 100 nm. The head diameter and tail length were 62.5±2.5 nm and 124.2±3.4 nm (mean ± SD nm; n = 12), respectively. (B) Structural proteins. The structural proteins were separated using a 12.5% SDS-PAGE gel. The N-terminal of the structural proteins (indicated by arrows and numbers) were sequenced. The protein sequences (numbered from 1 to 3) were SNFTAPVTTPSIPTPIQFLQ, MFQKQVYRQYTPGFPGDLIE, and MINISAFGSIVQFTASRTFP, respectively. The proteins numbered from 1 to 3 were identified as ORF23, ORF22, and ORF30, respectively. (C) Phylogenetic analysis based on the major structural proteins of PB1-like phage. Phage KPP12 was closely related to phage LMA2 of the PB1-like viruses. (D) Stability. After iodixanol density gradient ultra-centrifugal purification, phage KPP12 was stored in the dark at 4°C for one month. Data are shown as mean values with SD (n = 3). The phage was fairly stable during the experimental period.

Morphological analysis showed that phage KPP12 was classified into the family *Myoviridae* morphotype A1 (Fig. 1A). Genome sequencing of phage KPP12 revealed the presence of 64,144 bp, which corresponded approximately to the 64 kbp estimated by pulsed-field gel electrophoresis. The sequencing of the genome was circularly connected, implying that phage KPP12 genome is a linear form with terminal redundancy. Eighty-eight ORFs were predicted but no tRNA gene (Table S1). Analysis of the ORF by BLASTp revealed that the ORFs of phage KPP12 frequently showed high similarity to those of PB1-like viruses, including *Pseudomonas* phages LMA2, LBL3, SN, JG024, 14-1, F8, and PB1. Phage KPP12 and these PB1-like viruses also showed high similarity in terms of whole genomic DNA sequence (Fig. S1). BLASTp analysis of the phage KPP12 ORFs against the phage PB1 ORFs with an e-value cut-off of 0.1 revealed that 93.2% (82 out of 88 ORFs) of phage KPP12 ORFs were similar to those of phage PB1. Thus, phage KPP12 was considered to be a member of the PB1-like viruses. Moreover, structural protein analysis (Fig. 1B) showed that the major structural protein was ORF23, which is considered to be a major capsid protein. Since major capsid proteins can generally be used for phylogenetic analysis [20], the relationship between phage KPP12 and PB1-like viruses was investigated. Phage KPP12 showed the closest relationship to phage LMP2 (Fig. 1C). PB1-like viruses have been used in phage therapy [21] and are considered to be lytic phages.

Next, the therapeutic eligibility of KPP12 against *P. aeruginosa* infections was examined *in vitro*. This was achieved by the following: (i) determination of the *in vitro* activity of the phage against clinical strains, (ii) *in silico* examination of therapeutic phage eligibility, and (iii) assessment of the stability of the phage during storage. Firstly, KPP12 showed a host spectrum of 52.6% (20/38) against various clinical isolates and seemed to have a broader host range (80%, 4/5) for *P. aeruginosa* strains isolated from ophthalmic infections (Table 1). Genomic analysis of phage KPP12 identified no genes related to drug resistance, pathogenicity or lysogenicity (Table S1). In addition, phage KPP12 was categorized into the PB1-like phage group, which includes the therapeutic phage 14-1 (Fig. S1, Table S2, and Fig. 1C) [21]. Thirdly, although the infectivity of some phages decreased over time during storage, phage KPP12 was stable at 4°C for one month, even after iodixanol density gradient ultracentrifugation (Fig. 1D).

On the basis of the characteristics described above, KPP12 was considered to be a possible candidate phage for therapeutic and was used in the following experiments.

### Effects of Bacteriophage on Disease Course, Bacterial load, and PMN Infiltration in *P. aeruginosa* Keratitis

**Figure 2:**
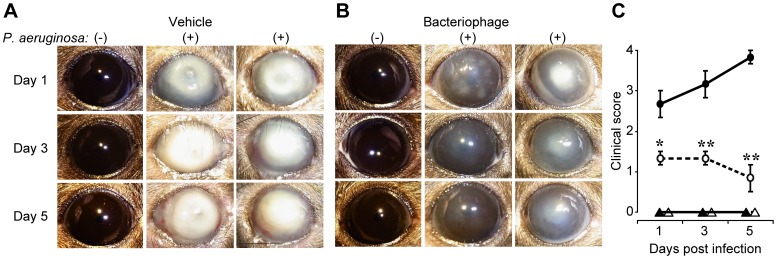
Effects of single-dose administration of topical phage KPP12 on the clinical signs of keratitis in infected mice. The scarified corneas were applied with 5.0×10^6^ CFU of *P. aeruginosa* or PBS as a control condition. Thirty minutes post-infection, 5 µl of vehicle (**A**) or bacteriophage (**B**) was administered topically. Representative photographs of vehicle- and bacteriophage-treated cornea on days 1, 3, and 5 p.i. (**C**) Clinical scores for corneal response in the vehicle-treated (closed circles) and bacteriophage-treated (open circles) mice assigned on days 1, 3, and 5 p.i. Clinical scores in vehicle- (closed triangles) and phage-treated (open triangles) mice without prior inoculation are shown. Data represent the mean +/− SEM of three experiments. *p<0.05, **P<0.01 (unpaired *t*-test) vs vehicle-treated mice.

Firstly, the effects of bacteriophage on non-infected corneal tissue were examined. The application of bacteriophage KPP12 or vehicle to the scarified cornea without prior inoculation induced no inflammatory responses, such as corneal opacity or inflammatory cell infiltrations (Fig. 2).

Secondly, a mouse model of keratitis was used to assess the ability of phage KPP12 eye-drops to kill *P. aeruginosa in vivo*. After infection of the cornea with *P. aeruginosa* PA33, the mice were treated with bacteriophage KPP12 eye-drops. Infected control mice received mock-treatment in the form of buffer without phages. A very severe clinical course was observed in mock-treated mice. A ring abscess was observed on day 1 p.i., and opacities had spread across the entire cornea by day 3 p.i. Most of the corneas perforated on day 5 p.i. (Fig. 2A). In contrast, PA33-infected mice treated with single-dose KPP12 eye-drops showed only slight or focal corneal opacities on day 1 p.i., and the corneal opacities gradually faded by day 5 p.i. (Fig. 2B). Treatment with KPP12 eye-drops resulted in a significantly improved disease outcome at days 1, 3, and 5 p.i., as indicated by the clinical scores (Fig. 2C).

**Figure 3:**
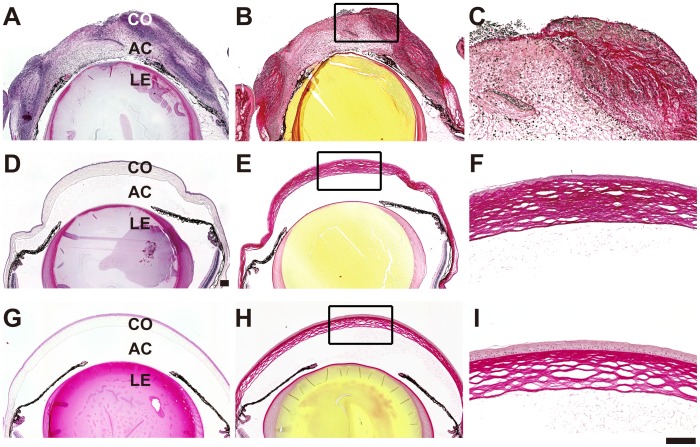
Histopathology of infected corneas treated with vehicle or bacteriophage. Representative photomicrographs of sections of the infected cornea on day 5 p.i. that were stained with H&E (A, D) or picrosirius red (B-C, E-F). Uninfected corneal sections stained with H&E (G) or picrosirius red (H-I) are shown as a control. In vehicle-treated mice (A-C), stromal collagen fibrils were severely damaged, resulting in central thinning and corneal stromal edema, and numerous inflammatory cells were observed. In phage-treated mice (D-F), normal corneal structure was maintained and no inflammatory cells were present. Bars: 100 µm. Co: cornea, AC: anterior chamber, L: lens.

**Figure 4:**
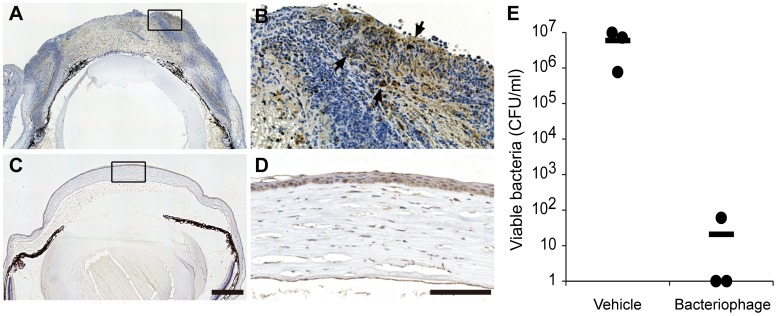
Bacterial clearance in bacteriophage-treated corneas. Representative photomicrographs of sections of infected cornea that were treated with vehicle (**A–B**) or bacteriophage KPP12 (**C–D**) at day 5 p.i. and stained with anti-P. aeruginosa Ab. P. *aeruginosa* within the abscess was stained brown (arrows) in mock-treated mice (**B**). **E**, on day 5 p.i., the corneas were excised and subjected to plate bacterial counting. Circles represent the data obtained from each mouse; horizontal bars represent mean values calculated from all mice in each group. *P<0.05 (Mann-Whitney *U* test) vs vehicle-treated mice. Bars: 100 µm.

Corneas from vehicle- and phage-treated mice were subjected to histopathological examination on day 5 p.i. The corneas of vehicle-treated mice exhibited denuded epithelium, central thinning and edema of the corneal stroma, and large neutrophilic abscesses. In mock-treated mice, picrosirius red staining revealed that the stromal structure of cornea was destroyed and that very few stromal collagen fibrils remained at the center of the cornea (Fig. 3B, C). In contrast, phage-treated mice showed an almost normal corneal structure (Fig. 3D, E, F). Examination of mAb-labeled histological sections revealed that *P. aeruginosa* was present within the abscess in mock-treated mice, whereas bacteria were barely detectable in phage-treated corneas. The bacterial load in the vehicle-treated mouse cornea was significantly higher at day 5 p.i. than in bacteriophage-treated mice (Fig. 4).

**Figure 5:**
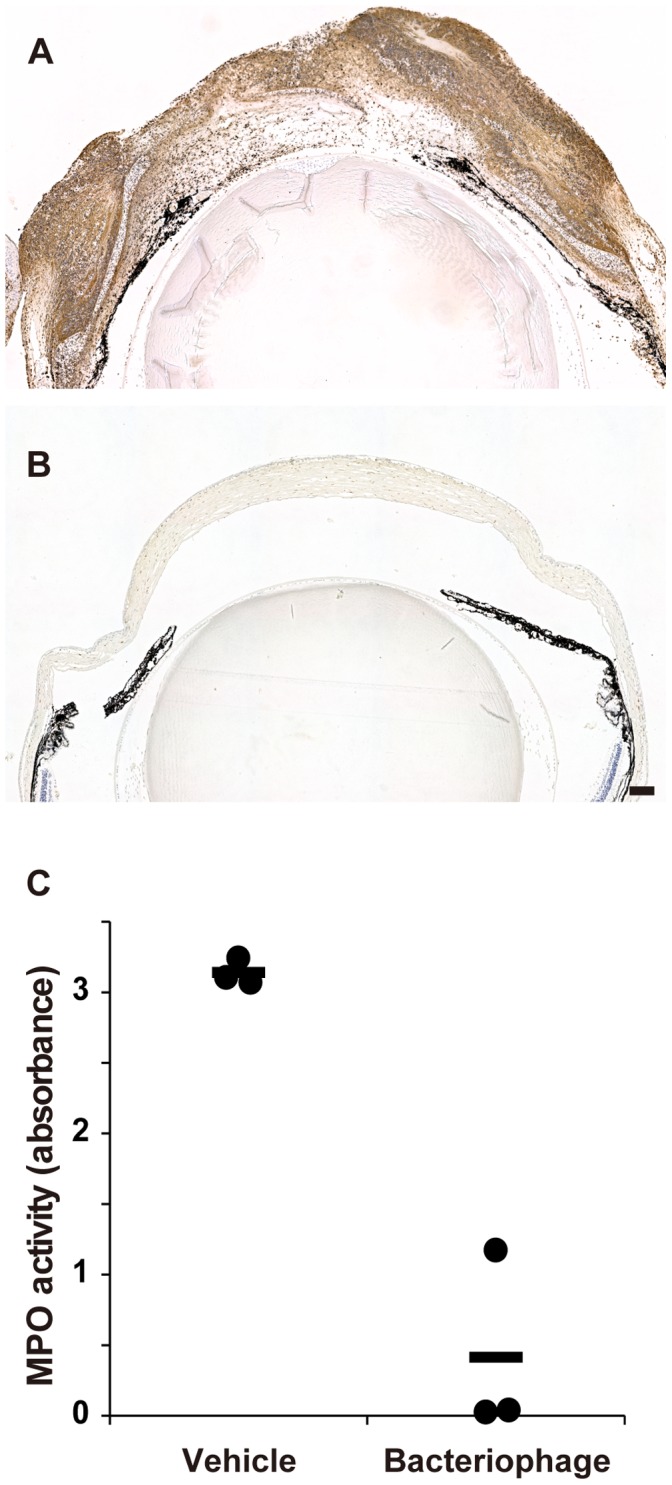
PMN infiltration in bacteriophage-treated corneal tissue. Representative photomicrographs of sections of infected cornea that were treated with vehicle (**A**) or bacteriophage KPP12 (**B**) on day 5 p.i. and stained with anti-Gr-1 Ab. C, on day 5 p.i., the corneas were excised and MPO activity was determined as a parameter of PMN infiltration. Circles represent the data obtained from each mouse; horizontal bars represent mean values calculated from all mice in each group. **P<0.01 (unpaired *t*-test) versus the corresponding value for vehicle-treated mice. Bar: 100 µm.

Immunostaining of neutrophils revealed the presence of numerous inflammatory cells in the cornea and the anterior chamber. An MPO assay to quantify PMN infiltration/persistence in the infected cornea revealed that the corneas of mock-treated mice contained significantly more PMN than those of bacteriophage-treated mice on day 5 p.i. (Fig. 5).

## Discussion

The present study demonstrated that the administration of a single-dose of bacteriophage KPP12 in the form of eye-drops efficiently eliminated bacteria from the infected cornea in a mouse model of *P. aeruginosa* keratitis. This resulted in improved disease outcome, preservation of the structural integrity and transparency of the cornea, and the suppression of neutrophils. These results suggest that bacteriophage eye-drops may represent a novel adjunctive or alternative therapy for antibiotic-resistant bacterial keratitis. To our knowledge, the present paper is the first to describe basic science research into bacteriophage therapy for ocular disease.

In terms of potential future clinical application, KPP12 appears to offer several important advantages. Firstly, KPP12 showed a broad host range for *P. aeruginosa* strains isolated from ophthalmic infections. Secondly, among the 88 predicted genes of phage KPP12, no genes related to drug resistance or pathogenicity were identified, and the genome does not contain a recognizable integrase gene, suggesting that this bacteriophage is lytic in nature. Thirdly, phage KPP12 was genetically related to the PB1-like phage group. DNA sequence analysis and structural protein analysis showed that KPP12 is a PB1-like virus (Fig. 1). Research suggests that PB-1 like viruses are lytic phages. In fact, a PB1-like virus (14-1) was selected for the treatment of burn-wounds in human clinical trials [21]. Fourthly, KPP12 could be stored at 4 °C for a period of one month with no decrease in infectivity, even after iodixanol density gradient ultracentrifugation. Finally, the phage can be administered in the form of eye-drops. The route of phage delivery appears to be a critical determinant of successful therapy [22]. In an acute respiratory infection model, phages were more effective when administered via intraperitoneal injection than via intranasal inhalation. This suggests that the phages may have been unable to penetrate the respiratory epithelium and encounter bacteria [22], [23]. The cornea is the outermost region of the eyeball, and the corneal epithelium is usually damaged at the site of infection in bacterial keratitis. Administration of the phage in the form of eye-drops may thus facilitate contact and infection of the bacteria present in the diseased cornea.

Although no basic science research into phage application in ocular disease has been published to date, two clinical studies have been reported [8], [11]. In 1970, Proskurov reported a good clinical outcome in 17 patients with conjunctivitis and blepharitis who were treated with anti-staphylococcal phage eye-drops [11]. However, no specific details concerning clinical course were reported. More recently, Slopek et al. reported the results of phage therapy in 550 patients with suppurative bacterial infection who presented at their institute between 1981 and 1986 [8]. Of these, good clinical outcome was reported in 16 patients with ocular infection, seven patients with purulent conjunctivitis, three patients with recurrent hordeolum, and one patient with dacryocystitis who had all been treated with phage eye-drops. Neither study reported any side effects to this treatment.

Another possible phage-related therapeutic agent is the bacterial cell-wall peptidoglycan-degrading enzyme lysin (endolysin), which is produced by the bacteriophage at the end stage of infection to allow the release of progeny virions. Phage endolysin is particularly effective against Gram-positive bacteria, because it specifically degrades the outermost peptidoglycan layer of these bacteria [24]. Recent studies have revealed that the exogenous administration of lysins extracted from certain phages was highly efficient in killing bacteria [25], [26], [27], [28]. The present authors previously reported the successful purification of a cloned lysin encoded by the *Staphylococcus aureus* bacteriophage φMR11. We found that this lysin efficiently lysed multidrug-resistant *S. aureus in vitro* and had therapeutic effects *in vivo* [29]. These findings strongly suggest the clinical usefulness of exogenous phage lysin for bacterial infectious diseases and that the ocular surface may be a suitable site for the delivery of lysin in the form of eye drops. In contrast to antibiotics, the cloned lysin, as with the phage itself, is highly specific to target bacteria but not to other members of the indigenous bacterial flora. The high specificity of lysin is an advantage in the clinical setting, since no substituted microbism can occur. Phage-related side-effects per se are uncommon, as neither phages nor their products (such as lysin) affect eukaryotic cells.

In conclusion, the present study has shown that KPP12 eye-drops may be a viable alternative therapy for *P. aeruginosa* keratitis in clinical practice. Since the incidence of bacterial keratitis is increasing due to inappropriate soft contact lens use and infection with multidrug-resistant bacteria, our results provide important fundamental data for future clinical studies into the use of phages on the ocular surface. As pointed out by Gorski et al, clinical trials are warranted to assess the therapeutic potential of phages in ocular disease, in particular in antibiotic-resistant cases [30].

## Supporting Information

Figure S1 **Multiple genomic alignments of phage KPP12 with PB1-like viruses.** The multiple genomic alignments were generated using Mauve software (http://gel.ahabs.wisc.edu/mauve/) and a progressive alignment with the default settings. The horizontal axis indicates the location of the genomes, and the vertical axis indicates the degree of DNA sequence similarity. The degree of similarity level is shown as percentage length (i.e., the higher bar indicates closer similarity). Phages are indicated on the right. A high degree of similarity was detected throughout the genomes of all phages. The degree of similarity declined around middle and terminal parts of the genome. The middle parts of the genomes showing lower similarity (i.e., 32–33.5 and 35–36.5 kbp in phage KPP) were considered to contain ORFs for tail proteins and DNA replication. The terminal parts of the genomes showing lower similarity were only seen sporadically and the function of their ORFs were not predictable. The genomic data of the PB1-like viruses were retrieved from the GenBank (phage 14-1, FM897211; phage F8, DQ163917; phage SN, FM887021; phage PB1, EU716414; phage LMA2, FM201282; phage LBL3, FM201281; phage JG024, GU815091).
(TIF)

Table S1 **Annotation of phage KPP12.**
(DOC)

Table S2 **Phages hit by BLASTp search based on the major capsid protein.**
(DOC)
