# Supplementary figures and images for: Pseudomonas aeruginosa Keratitis in Mice: Effects of Topical Bacteriophage KPP12 Administration
Source: PLoS One. 2012 Oct 17;7(10):e47742. doi: 10.1371/journal.pone.0047742 (PMC3474789; doi:10.1371/journal.pone.0047742)

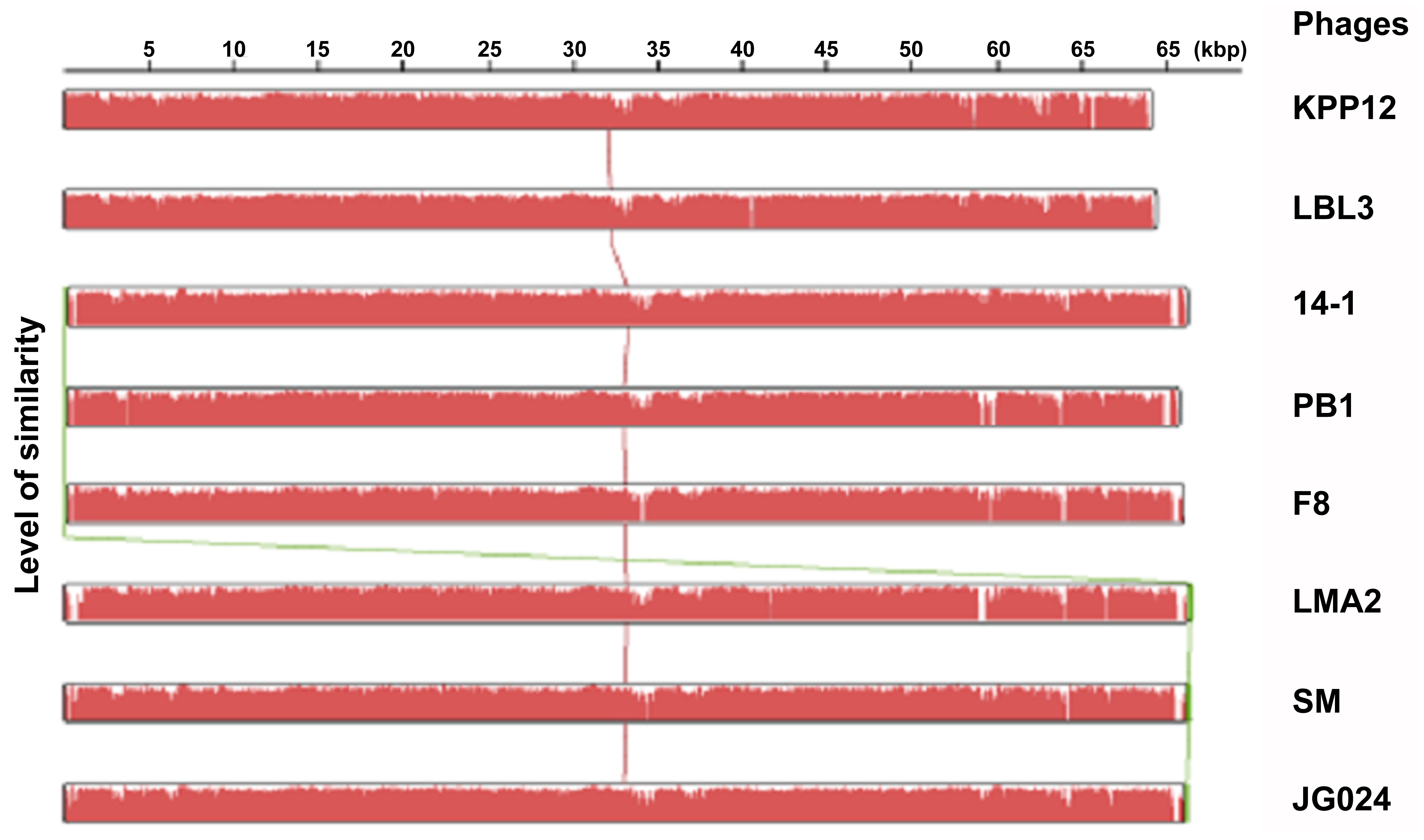

Supplement: Figure S1 — Multiple genomic alignments of phage KPP12 with PB1-like viruses. The multiple genomic alignments were generated using Mauve software (http://gel.ahabs.wisc.edu/mauve/) and a progressive alignment with the default settings. The horizontal axis indicates the location of the genomes, and the vertical axis indicates the degree of DNA sequence similarity. The degree of similarity level is shown as percentage length (i.e., the higher bar indicates closer similarity). Phages are indicated on the right. A high degree of similarity was detected throughout the genomes of all phages. The degree of similarity declined around middle and terminal parts of the genome. The middle parts of the genomes showing lower similarity (i.e., 32–33.5 and 35–36.5 kbp in phage KPP) were considered to contain ORFs for tail proteins and DNA replication. The terminal parts of the genomes showing lower similarity were only seen sporadically and the function of their ORFs were not predictable. The genomic data of the PB1-like viruses were retrieved from the GenBank (phage 14-1, FM897211; phage F8, DQ163917; phage SN, FM887021; phage PB1, EU716414; phage LMA2, FM201282; phage LBL3, FM201281; phage JG024, GU815091). (TIF) [file pone.0047742.s001.tif]
