# Supplementary material for: Pseudomonas aeruginosa Keratitis in Mice: Effects of Topical Bacteriophage KPP12 Administration
Source: PLoS One. 2012 Oct 17;7(10):e47742. doi: 10.1371/journal.pone.0047742 (PMC3474789; doi:10.1371/journal.pone.0047742)
Supplement: Table S1 — Annotation of phage KPP12. (DOC) [file pone.0047742.s002.doc]

**Table S1. Annotation of phage KPP12**

|  | **Direction** | **Left end** | **Right end** | **Length (nt)** | **Length (AA)** | **Putative function** |
| --- | --- | --- | --- | --- | --- | --- |
| **ORF1** | + | 149 | 1531 | 1383 | 460 | terminase |
| **ORF2** | - | 569 | 1282 | 714 | 237 |  |
| **ORF3** | - | 1568 | 1951 | 384 | 127 |  |
| **ORF4** | - | 1948 | 2166 | 219 | 72 |  |
| **ORF5** | - | 2166 | 2516 | 351 | 116 |  |
| **ORF6** | - | 2562 | 2960 | 399 | 132 |  |
| **ORF7** | - | 2963 | 3742 | 780 | 259 |  |
| **ORF8** | - | 3829 | 4266 | 438 | 145 |  |
| **ORF9** | - | 4283 | 4870 | 588 | 195 |  |
| **ORF10** | - | 4880 | 4975 | 96 | 31 |  |
| **ORF11** | - | 4972 | 5901 | 930 | 309 |  |
| **ORF12** | - | 6005 | 6352 | 348 | 115 |  |
| **ORF13** | + | 6490 | 6657 | 168 | 55 |  |
| **ORF14** | - | 6601 | 6912 | 312 | 103 |  |
| **ORF15** | - | 7118 | 7441 | 324 | 107 |  |
| **ORF16** | - | 7473 | 7874 | 402 | 133 |  |
| **ORF17** | + | 8055 | 10352 | 2298 | 765 | Minor head protein |
| **ORF18** | + | 10352 | 11188 | 837 | 278 | Minor head protein |
| **ORF19** | + | 11207 | 11413 | 207 | 68 |  |
| **ORF20** | + | 11410 | 11550 | 141 | 46 |  |
| **ORF21** | + | 12063 | 13496 | 1434 | 477 | Structural protein |
| **ORF22** | + | 13500 | 14135 | 636 | 211 | Structural protein |
| **ORF23** | + | 14145 | 15293 | 1149 | 382 | Major structural protein |
| **ORF24** | + | 15395 | 15832 | 438 | 145 |  |
| **ORF25** | + | 15847 | 16314 | 468 | 155 | Structural protein |
| **ORF26** | + | 16311 | 16709 | 399 | 132 | Structural protein |
| **ORF27** | + | 16717 | 17268 | 552 | 183 |  |
| **ORF28** | + | 17265 | 17846 | 582 | 193 |  |
| **ORF29** | + | 17862 | 19376 | 1515 | 504 | Structural protein |
| **ORF30** | + | 19435 | 19887 | 453 | 150 | Structural protein |
| **ORF31** | + | 19887 | 20210 | 324 | 107 | Structural protein |
| **ORF32** | + | 20207 | 20989 | 783 | 260 | Structural protein |
| **ORF33** | + | 20999 | 21502 | 504 | 167 | Structural protein |
| **ORF34** | + | 21502 | 22041 | 540 | 179 | Structural protein |
| **ORF35** | + | 22050 | 22643 | 594 | 197 | Structural protein |
| **ORF36** | + | 22653 | 23081 | 429 | 142 |  |
| **ORF37** | + | 23085 | 25661 | 2577 | 858 | Lytic tail protein |
| **ORF38** | + | 25661 | 26524 | 864 | 287 | Structural protein |
| **ORF39** | + | 26524 | 27057 | 534 | 177 |  |
| **ORF40** | + | 27113 | 27778 | 666 | 221 | Baseplate |
| **ORF41** | + | 27835 | 29088 | 1254 | 417 | Baseplate |
| **ORF42** | + | 29085 | 30599 | 1515 | 504 | Structural protein |
| **ORF43** | + | 30604 | 33495 | 2892 | 963 | Tail protein |
| **ORF44** | + | 33497 | 33925 | 429 | 142 | Tail fiber component |
| **ORF45** | + | 33925 | 34587 | 663 | 220 | Endolysin |
| **ORF46** | - | 34612 | 34863 | 252 | 83 |  |
| **ORF47** | - | 35144 | 36055 | 912 | 303 | Ligase |
| **ORF48** | - | 36110 | 36664 | 555 | 184 |  |
| **ORF49** | - | 36661 | 37266 | 606 | 201 |  |
| **ORF50** | - | 37320 | 38219 | 900 | 299 |  |
| **ORF51** | - | 38308 | 38928 | 621 | 206 |  |
| **ORF52** | - | 39023 | 40582 | 1560 | 519 | Helicase |
| **ORF53** | - | 40579 | 40989 | 411 | 136 |  |
| **ORF54** | - | 40982 | 44089 | 3108 | 1035 | DNA polymerase |
| **ORF55** | - | 44089 | 44643 | 555 | 184 | DNA polymerase |
| **ORF56** | - | 44719 | 45735 | 1017 | 338 | Polynucleotide kinase |
| **ORF57** | - | 45738 | 45947 | 210 | 69 |  |
| **ORF58** | - | 45931 | 46848 | 918 | 305 | Thymidylate synthase |
| **ORF59** | - | 46848 | 47054 | 207 | 68 |  |
| **ORF60** | - | 47062 | 47289 | 228 | 75 |  |
| **ORF61** | - | 47322 | 47540 | 219 | 72 |  |
| **ORF62** | - | 47524 | 47742 | 219 | 72 |  |
| **ORF63** | - | 47742 | 47972 | 231 | 76 |  |
| **ORF64** | - | 48060 | 49061 | 1002 | 333 |  |
| **ORF65** | - | 49166 | 50056 | 891 | 296 | Structural protein |
| **ORF66** | - | 50217 | 51404 | 1188 | 395 | Exonuclease |
| **ORF67** | - | 51391 | 51813 | 423 | 140 |  |
| **ORF68** | + | 51981 | 52766 | 786 | 261 | Endonuclease |
| **ORF69** | + | 52776 | 53690 | 915 | 304 |  |
| **ORF70** | + | 53690 | 54139 | 450 | 149 |  |
| **ORF71** | + | 54136 | 55212 | 1077 | 358 |  |
| **ORF72** | + | 55218 | 55403 | 186 | 61 |  |
| **ORF73** | + | 55551 | 57290 | 1740 | 579 | Primase |
| **ORF74** | + | 57488 | 58117 | 630 | 209 |  |
| **ORF75** | + | 58459 | 58866 | 408 | 135 |  |
| **ORF76** | - | 59033 | 59647 | 615 | 204 |  |
| **ORF77** | - | 59839 | 60510 | 672 | 223 |  |
| **ORF78** | - | 60515 | 60754 | 240 | 79 |  |
| **ORF79** | - | 60762 | 61073 | 312 | 103 |  |
| **ORF80** | - | 61123 | 61344 | 222 | 73 |  |
| **ORF81** | - | 61400 | 61624 | 225 | 74 |  |
| **ORF82** | - | 62009 | 62674 | 666 | 221 |  |
| **ORF83** | - | 62706 | 62918 | 213 | 70 |  |
| **ORF84** | - | 62915 | 63139 | 225 | 74 |  |
| **ORF85** | - | 63136 | 63351 | 216 | 71 |  |
| **ORF86** | - | 63348 | 63548 | 201 | 66 |  |
| **ORF87** | - | 63545 | 63796 | 252 | 83 |  |
| **ORF88** | - | 63964 | 64122 | 159 | 52 |  |
