# Supplementary material for: Pseudomonas aeruginosa Keratitis in Mice: Effects of Topical Bacteriophage KPP12 Administration
Source: PLoS One. 2012 Oct 17;7(10):e47742. doi: 10.1371/journal.pone.0047742 (PMC3474789; doi:10.1371/journal.pone.0047742)
Supplement: Table S2 — Phages hit by BLASTp search based on the major capsid protein. (DOC) [file pone.0047742.s003.doc]

**Table S2. Phages hit by BLASTp search based on the major capsid protein**

|  |  |  | **Genome** | |  |
| --- | --- | --- | --- | --- | --- |
| **Host bacteria** | **Phage** | **Family** | **Size (bp)** | **G+C content (%)** | **GenBank** |
| *Pseudomonas aeruginosa* | *Pseudomonas* phage JG024 | *Myoviridae* | 66,275 | 55 | GU815091 |
| *Pseudomonas aeruginosa* | *Pseudomonas* phage PB1 | *Myoviridae* | 65,764 | 54 | [EU716414](http://www.ncbi.nlm.nih.gov/nuccore/EU716414) |
| *Pseudomonas aeruginosa* | *Pseudomonas* phage F8 | *Myoviridae* | 66,015 | 54 | [DQ163917](http://www.ncbi.nlm.nih.gov/nuccore/DQ163917) |
| *Pseudomonas aeruginosa* | *Pseudomonas* phage LBL3 | *Myoviridae* | 64,427 | 55 | [FM201281](http://www.ncbi.nlm.nih.gov/nuccore/FM201281) |
| *Pseudomonas aeruginosa* | *Pseudomonas* phage SN | *Myoviridae* | 66,390 | 55 | [FM887021](http://www.ncbi.nlm.nih.gov/nuccore/FM887021) |
| *Pseudomonas aeruginosa* | *Pseudomonas* phage 14-1 | *Myoviridae* | 66,235 | 55 | [FM897211](http://www.ncbi.nlm.nih.gov/nuccore/FM897211) |
| *Pseudomonas aeruginosa* | *Pseudomonas* phage LMA2 | *Myoviridae* | 66,530 | 55 | [FM201282](http://www.ncbi.nlm.nih.gov/nuccore/FM201282) |
| *Burkholderia cenocepacia* | *Burkholderia* phage BcepB1A | *Myoviridae* | 47,399 | 54 | [AY616033](http://www.ncbi.nlm.nih.gov/nuccore/AY616033) |
| *Burkholderia cenocepacia* | *Burkholderia* phage BcepNY3 | *Myoviridae* | 47,382 | 63 | [EF602154](http://www.ncbi.nlm.nih.gov/nuccore/EF602154) |
| *Burkholderia cenocepacia* | *Burkholderia* phage Bcep1 | *Myoviridae* | 48,177 | 63 | [AY369265](http://www.ncbi.nlm.nih.gov/nuccore/AY369265) |
| *Burkholderia cepacia* | *Burkholderia* phage Bcep43 | *Myoviridae* | 48,024 | 63 | [AY368235](http://www.ncbi.nlm.nih.gov/nuccore/AY368235) |
| *Burkholderia cepacia* | *Burkholderia* phage Bcep781 | *Myoviridae* | 48,247 | 63 | [AF543311](http://www.ncbi.nlm.nih.gov/nuccore/AF543311) |
| *Xanthomonas oryzae* | *Xanthomonas* phage OP2 | ?? | 46,643 | 60 | [AP008986](http://www.ncbi.nlm.nih.gov/nuccore/AP008986) |
| *Burkholderia ambifaria* | *Burkholderia* phage BcepF1 | *Myoviridae* | 72,415 | 55 | [EF153632](http://www.ncbi.nlm.nih.gov/nuccore/EF153632) |
